# Automated classification of coronary plaque calcification in OCT pullbacks with 3D deep neural networks

**DOI:** 10.1117/1.JBO.25.9.095003

**Published:** 2020-09-10

**Authors:** Chunliu He, Jiaqiu Wang, Yifan Yin, Zhiyong Li

**Affiliations:** a Southeast University, School of Biological Science and Medical Engineering, Nanjing, China; b Queensland University of Technology, School of Mechanical, Medical and Process Engineering, Brisbane, Queensland, Australia

**Keywords:** atherosclerosis, plaque calcification, intravascular optical coherence tomography, deep learning

## Abstract

**Significance:** Detection and characterization of coronary atherosclerotic plaques often need reviews of a large number of optical coherence tomography (OCT) imaging slices to make a clinical decision. However, it is a challenge to manually review all the slices and consider the interrelationship between adjacent slices.

**Approach**: Inspired by the recent success of deep convolutional network on the classification of medical images, we proposed a ResNet-3D network for classification of coronary plaque calcification in OCT pullbacks. The ResNet-3D network was initialized with a trained ResNet-50 network and a three-dimensional convolution filter filled with zeros padding and non-zeros padding with a convolutional filter. To retrain ResNet-50, we used a dataset of ∼4860 OCT images, derived by 18 entire pullbacks from different patients. In addition, we investigated a two-phase training method to address the data imbalance. For an improved performance, we evaluated different input sizes for the ResNet-3D network, such as 3, 5, and 7 OCT slices. Furthermore, we integrated all ResNet-3D results by majority voting.

**Results**: A comparative analysis proved the effectiveness of the proposed ResNet-3D networks against ResNet-2D network in the OCT dataset. The classification performance (F1-scores=94% for non-zeros padding and F1-score=96% for zeros padding) demonstrated the potential of convolutional neural networks (CNNs) in classifying plaque calcification.

**Conclusions**: This work may provide a foundation for further work in extending the CNN to voxel segmentation, which may lead to a supportive diagnostic tool for assessment of coronary plaque vulnerability.

## 1 Introduction

Coronary artery calcification (CAC) is associated with major adverse cardiovascular events.^1^^–^^3^ To date, the clinical impact of arterial calcification on local plaque vulnerability remains unclear,^4^ but the extent of CAC is associated with worse outcomes in the general population and in patients undergoing revascularization.^5^^,^^6^ In addition, the presence of calcifications leads to under-expansion during percutaneous coronary intervention (PCI).^7^ The previous study demonstrated that severely calcified plaques undergoing PCI were associated with higher procedural complications and lower success rates.^8^ Hence, detection of calcified region is important for PCI treatment. For example, in the heavily calcified lesions, it is hard to cross or dilate a coronary stenosis with PCI devices such as balloons or stents.^9^ However, rotational atherectomy facilitates delivery or expansion for the treatment of complex calcified lesions. Furthermore, other available treatment strategies such as cutting and scoring balloons improve vessel compliance by creating discrete incisions instead of producing small particles to ablate the heavily calcified plaque for rotablation.^4^ For above reasons, a cardiologist pays more attention to the calcified lesion when making a clinical decision. Therefore, accurate identification and quantification of calcified lesion is crucial when treating patients with advanced coronary atherosclerosis.

The quantitative analysis of calcified lesions is a challenging task for a number of reasons. The heterogeneous appearance of the lesions including the large variability in location, size, shape, and frequency makes it difficult to conduct effective and quantitative analysis.^10^ An imaging system containing artifacts such as speckle, motion artifacts makes the image interpretation challenging to a novice reader. Manual detection is the current approach, but is tedious, expensive, time-consuming, impractical in larger studies, and it introduces interobserver variability. Moreover, the lesion is intrinsically a 3D structure and a single slice measurement is unable to characterize the volumetric nature of the lesion. To decide a particular tissue component, a multiple image slices need to be considered. Moreover, the level of expert knowledge and experience is also an important factor that affects the accuracy. Hence, in clinical routine often only qualitative, visual inspection, or at best crude measures such as approximate lesion volume are used.^11^^–^^13^ Therefore, development of an accurate, automatic plaque classification method is needed to preferentially provide a feedback on the existence of calcification deposits on the pullback level.

Both intravascular optical coherence tomography (OCT) and intravascular ultrasound (IVUS) can be used to detect a calcified plaques.^14^^,^^15^ OCT has been proven to be a primary choice because of its high resolution (10 to 15  μm) in comparison with IVUS.^16^ Although IVUS has been widely used to estimate coronary calcification during PCI to date,^17^ it is not able to precisely visualize microcalcification. Moreover, IVUS cannot penetrate heavily calcified plaques in the assessment of coronary calcification. In this study, we developed a 3D convolutional neural network (CNN) for automatic classification of calcification lesion on the OCT pullback level. Full fine-tuning, a pretrained ResNet-2D network allowed us to transfer natural image features to OCT images. Furthermore, we also compared different input slices to evaluate the ResNet-3D network performance. Finally, a majority voting to integrate all the ResNet-3D networks was used to further improve the classification results.

## 2 Related Works

Various studies have assessed the efficacy of the quantification and characterization of calcified plaques using machine learning based on OCT images.^18^^–^^20^ Most of these researches have emphasized segmentation of the calcified plaques using the traditional machine learning methods on a single OCT slice. The atherosclerotic plaque segmentation has not been adequately and fully performed because it needs to manually select the slices with a calcified deposit in advance. For this reason, plaque segmentation should be the next important step, and the feedback on the existence and characteristics of calcium deposits on the pullback level should be the priority.^21^ Furthermore, deep learning has once again become a state-of-the art learning algorithm in the image classification. Compared to the traditional machine learning, deep networks naturally integrate low-, mid-, and high-level image features.^22^ Machine learning focuses on mining prior knowledge in the data and converting it to corresponding regularization constraints or artificial design features. Deep learning is weak on the prior knowledge of the data. It is expected to learn the data through stacking of hierarchical expressions. The intrinsic regularity of this makes this kind of separability or discriminative feature as much as possible to weaken the complex design of the classifier and achieve the purpose of simplicity, novelty, and universality. Recently, there has been increasingly more attention on deep learning based on the intravascular OCT images for detection and characterization atherosclerotic plaque. Abdolmanafi et al.^23^^–^^25^ studied the CNN application in the OCT slices and confirmed the feasibility and effectiveness of deep learning in detection and characterization of atherosclerotic plaque. They successfully classified the coronary artery layers in pediatric patients using the powerful feature representation abilities of CNN models. In addition, they also compared three common deep learning models (e.g., Alexnet, VGG-19, and Inception-v3) for tissue characterization of intracoronary pathological formations. The results demonstrated that deep learning models are robust for automatic interpretation of the OCT slices. Furthermore, they designed a diagnostic model about coronary artery lesions using multisteps strategies based on the above studies. These studies demonstrated successful applications of the pretrained CNN networks on OCT dataset. However, it had shown that a custom network could obtain a more effective and robust result in the characterization of OCT slices. Kolluru et al.^26^ classified three tissue types (fibrocalcific, fibrolipidic, or others) by A-line using the customized CNN and fully connected artificial neural network. In addition, the hybrid model such as the restructure pretrained model based on the specific task could improve the network performance. Gessert et al.^21^ executed a multipath network architecture based on the ResNet network and achieved an accuracy of 91.7%, a sensitivity of 90.9%, and a specificity of 92.4%. Lee et al.^27^ developed a fully automated semantic segmentation model of atherosclerotic plaques for OCT images. The sensitivities/specificities against manually annotated clinical dataset were 87.4%/89.5% and 85.1%/94.2% for pixelwise classification of lipid and calcified plaque, respectively. They also used hybrid features, including the deep features, handcrafted features and lumen morphological features to characterize atherosclerotic plaque in OCT image. All the above studies were based on a single OCT slice, which reduced the training time but limited the further performance improvement. Even though single OCT slice provided the local and global information of the plaque, considering the fact that a coronary lesion is intrinsically a 3D structure, 3D CNN may be a better approach.

One important aspect of CNNs is the “transferability” of knowledge embedded in the pretrained CNNs. Recent research conducted by Tajbakhsh et al.^28^ consistently demonstrated that fine-tuning was better than trained from scratch for deep networks. Moreover, layerwise fine-tuning is an alternative optimal choice to reach the better performance for the limited medical image dataset. Several studies have demonstrated that a pretrained CNN could be adapted to the medical image classification.^29^^–^^31^ For instance, Christodoulidis et al.^30^ fine-tuned different CNN layers for classification of interstitial lung diseases. Their studies suggested that the ensemble learn could help for the unbalance medical images. In another study, two pretrained networks, VGG 16 and Inception V4, were employed to class the Alzheimer’s disease from MRI images.^31^ The labeled MRI data were used to train the fully connected layers while keeping the rest network layer fixed. Different from the previous approaches, Miyagawa et al.^29^ transferred the knowledge from other tasks based on OCT images to the classification of vascular bifurcation, and this method yielded promising results.

## 3 Methods

**Fig. 1:**
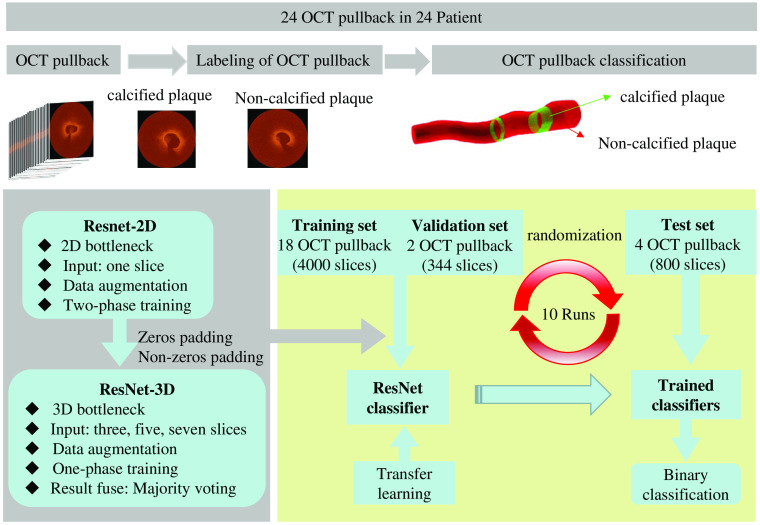
Flowchart of the methodology. 2D, two dimension; 3D: three dimension.

**Fig. 2:**
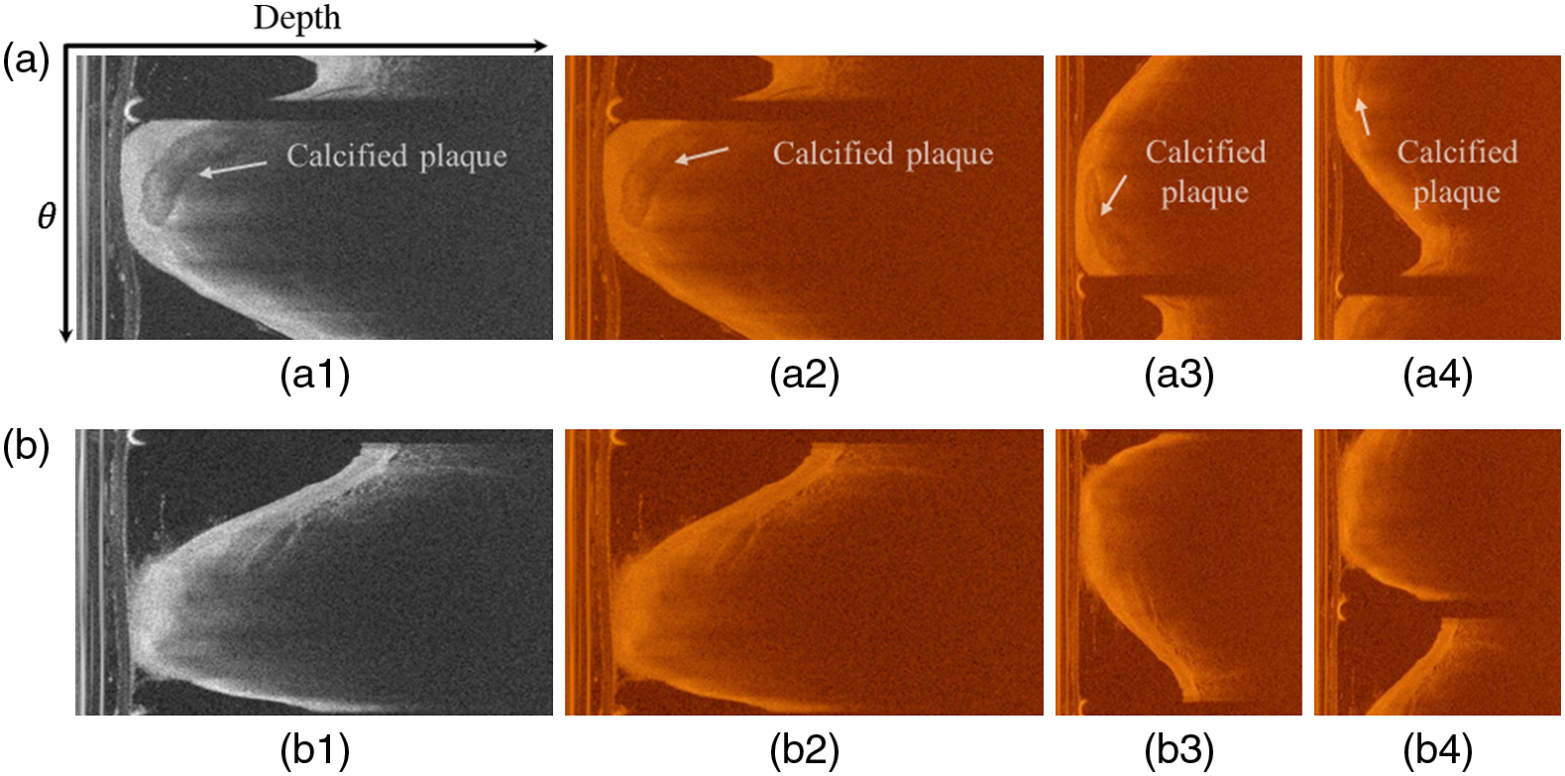
The polar representations of OCT images. (a) Preprocessed calcified image, (b) preprocessed noncalcified image. (a1, b1) gray image, (a2, b2) RGB image, (a3, b3) augmentation image with flip along the depth axis, and (a4, b4) augmentation image with cycle translation along the depth axis.

Figure 1 shows the flowchart of the methodology. A trained expert reviewed each pullback independently and labeled each slice with calcified plaque and noncalcified plaque. According the expert consensus, calcium components are well-delineated heterogeneous regions in the arterial wall with less signal attenuation,^32^ as can be seen in Fig. 2.

### 3.1 OCT Dataset

The OCT images were acquired using a commercially available Fourier Domain OCT system (2.7F C7-XR, St. Jude Medical, St. Paul, Minnesota) and Dragonfly catheter (St. Jude). A total of 24 patients were taken from Affiliated Drum Tower Hospital, Nanjing University between December 2016 and December 2018. All participants provided a written informed consent prior to the enrollment, and the study protocol was approved by the institutional ethics committee.

It is worth noting that each patient had one pullback, and each pullback contained unequal number of images. All images of each pullback were used in this study. The training set consisted of 18 pullbacks, in which 2 pullbacks were for validation and the remaining 4 pullbacks were the test set (Fig. 1). Each experiment repeated 10 runs for different pullbacks, and the means were reported as the experimental results. The quantification of evaluation criteria was calculated based on the test set.

### 3.2 OCT Image Preprocessing

The polar representation was used in the study. The original polar image in this study had a resolution of 504×976. Given the input size of ResNet-2D network, we resized the images to a resolution of 256×256. It is important to note that the input of ResNet-2D network needs color images, thus we transferred the gray polar images to color images as shown in Fig. 2. According to the input size of ResNet-3D networks, an original pullback dataset can be expended as

$$X_{\text{expand}}=\{x_{1}^{c},x_{1},x_{2},\ldots ,x_{N},x_{N}^{c}\},$$ (1)

$$c=\{\begin{array}{cc} 1 & d=3\\ 2 & d=5\\ 3 & d=7 \end{array},$$ (2)

where $x_{1},x_{2},,\ldots ,x_{N}$ are the OCT slices in one pullback, N is the total number of images in a pullback, c is the number of slices that needs to be expanded, and d is the number of slices.

Data augmentation is considerably important for increasing dataset, strengthening the generalization capability of the model, and preventing overfitting.^33^ Given the image characteristics of polar representation, two effective data augmentation, cycle translation with 1-256 pixels, and flip along the depth axis were used (see Fig. 2). For the ResNet-2D networks, data augmentations were applied to the single OCT slice. In case of 3D CNNs, each volume was processed as a single entity using the same data augmentations.

### 3.3 Convolutional Neural Networks

#### 3.3.1 Network architectures

**Fig. 3:**
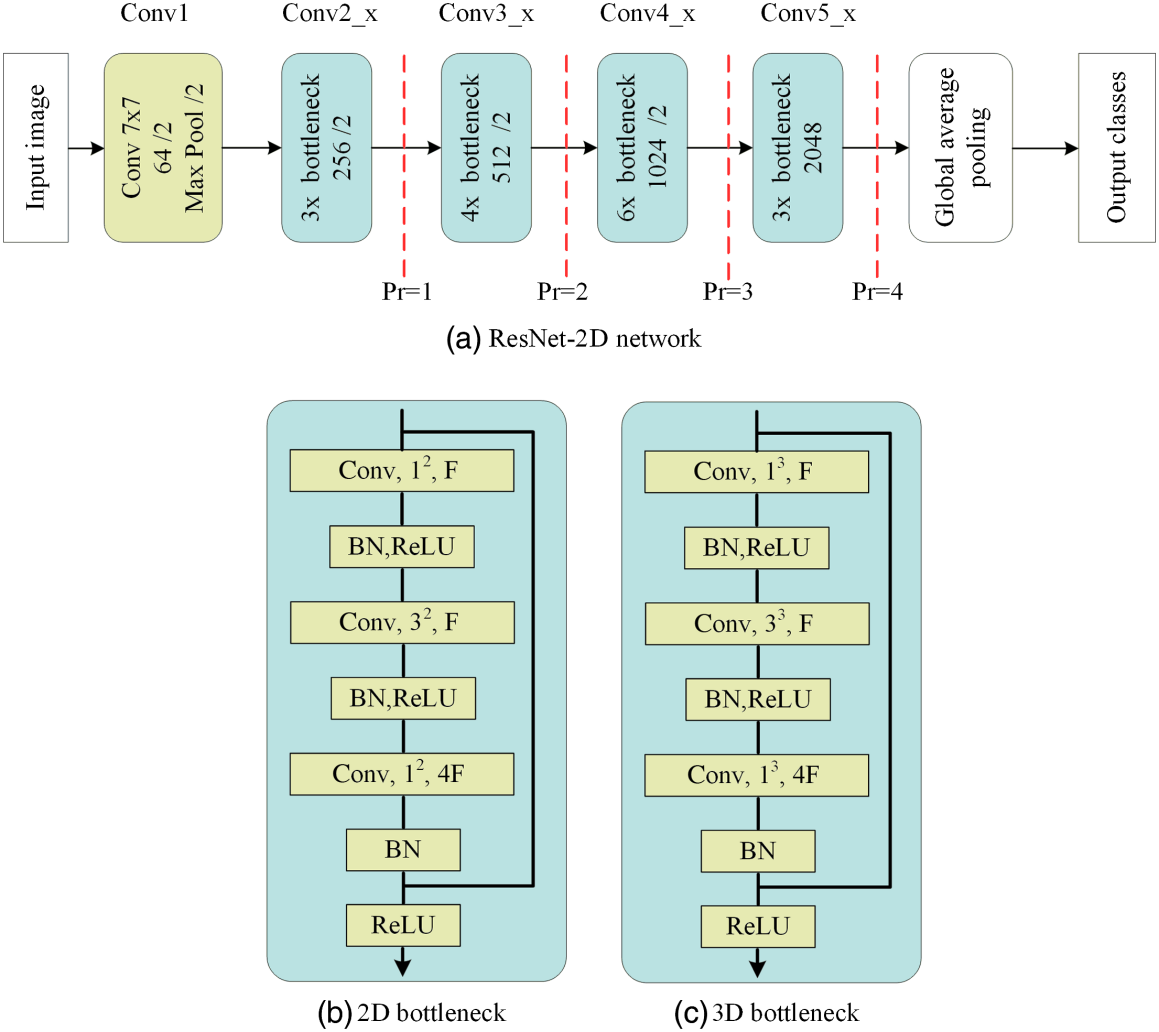
The network architectures that were employed with our dataset. In each ResNet input and output block, the number of output feature maps was given. /2 denotes spatial downsampling with a stride of two. pr denotes a retraining point for transfer learning. Left of the retraining point, weights are freezed, right of the retraining point, weights are retrained.

We employed one of state-of-the-art architectures from the natural image domain, 50-layer ResNet-2D as an initial network model in the experiment. The 50-layer ResNet-2D has an obvious hierarchical structure and powerful output with representational ability. In addition, a large amount of downsampling was used, which significantly improved the gradient propagation efficiency. Batch normalization and global average pooling for regularization speeded up the training of the network. Furthermore, the network utilized the bottlenecks to reduce network parameters. Here, a 1×1 convolution first downsampled and then upsampled the tensor along the feature map dimension. Then, the normal 3×3 filter embedded on the lower input/output dimension. The 2D bottleneck is shown in Fig. 3(b). To realize the ResNet-3D architecture, we directly expended the 2D bottleneck to the 3D bottleneck [Fig. 3(c)]. Both main architectures were the same, whereas the convolution kernel of the bottleneck part was different. The basic bottleneck block in the Resnet-3D consisted of three convolutional layers, and the kernel sizes of the first and the third convolutional layers were 1×1×1, whereas it was 3×3×3 for the second layer.

#### 3.3.2 Loss function

It is important to choose an appropriate loss function on the pullback level. With the rise of deep learning, cross entropy loss function becomes a recognized effective loss function, especially in the field of image classification.^34^ Assume that $X_{1},\ldots ,X_{N1}$ is a set of N1 training samples and $y_{1},\ldots ,y_{n}\in \{1,\ldots ,M\}$ (M is the class number) are the corresponding class labels. Cross entropy loss function is defined as

$$L_{i}=\overset{M}{\underset{j=1}{\sum }}y_{i,j}\;\log(p_{i,j}),$$ (3)

where $y_{i,j}$ is the number of the class, and $y_{i,j}$ is 1 if the sample $X_{i}$ belongs to class $y_{i}$ ($j=y_{i}$) and 0 otherwise. $p_{i,j}$ is the class probabilities that is normalized by the softmax function. Cross entropy loss with softmax function is commonly used as the output layer, which could provide a classification probability distribution.

#### 3.3.3 Training

The ResNet-2D network was trained on the ImageNet dataset, consisting of 1.2 million training images, with 1000 classes of objects. It is unwise to directly apply the pretrained ResNet-2D network to the OCT datasets because of the significant differences between nature images and OCT images. Therefore, we replaced image mean of the pretrained network with OCT images before the retraining started using the OCT dataset in our ResNet-2D models. Then, we simultaneously set the fully connected layer to have the same size as the number of classes in the OCT dataset. Accordingly, we also replaced the final layer with a new classification output layer where the classes were default to automation. To further investigate the effects of different transfer learning strategies, we accurately froze different weights layers. Compared to the ResNet-2D models, the training of ResNet-3D models is intuitively more difficult because of more parameters and expensive computational time. With the availability of the trained ResNet-2D model, it is a natural choice to utilize the trained ResNet-2D model parameters to initialize the ResNet-3D models. However, the sizes of both convolution kernels often do not match. In order to solve the problem, we assume $H\in R^{c_{\text{in}}\times c_{\text{out}}\times 3\times 3}$ is the trained ResNet-2D network convolutional filter where $C_{\text{in}}$ and $C_{\text{out}}$ are the channel numbers of the input and output, respectively. According to the assumption, we implemented non-zeros padding and zeros padding to pad the 3D convolution filter $D\in R^{c_{\text{in}}\times c_{\text{out}}\times 3\times 3\times 3}$ as

$$D_{\text{non-zeros padding}}=\{\begin{array}{l} D_{\left(0\right)}=H_{C_{\text{in}}\times C_{\text{out}}\times 3\times 3}\\ D_{\left(1\right)}=H_{C_{\text{in}}\times C_{\text{out}}\times 3\times 3}\\ D_{\left(2\right)}=H_{C_{\text{in}}\times C_{\text{out}}\times 3\times 3} \end{array},$$ (4)

$$D_{\text{zeros padding}}=\{\begin{array}{c} D_{\left(0\right)}=0_{C_{\text{in}}\times C_{\text{out}}\times 3\times 3}\\ D_{\left(1\right)}=H_{C_{\text{in}}\times C_{\text{out}}\times 3\times 3}\\ D_{\left(2\right)}=0_{C_{\text{in}}\times C_{\text{out}}\times 3\times 3} \end{array},$$ (5)

where the subscript of D is the depth of ResNet-3D convolution. By design, the initialized ResNet-3D model is based on a trained ResNet-2D model, which may achieve at least the same performance as the ResNet-2D model.

Training with original imbalance dataset makes it impossible for deep network to favor minority class through entire pullbacks. To overcome the difficulty, a two-phase training method was implemented. In the first phase training, the labels of the dataset were rigorously equiprobable by oversampling. In the second phase, the original and imbalance data were used to fine-tune the output layer and keep the kernels of all others fixed.

In the study, we used the Adam algorithm^35^ with a starting learning rate of $10^{-4}$. To find the optimal schedule, we reduced the learning rate by a factor of two when the validation error saturated. A batch size of Batchnorm = 64 was used for all models. In addition, early stop of the training could also happen when the classification accuracy on the validation set did not improve. All analyses were conducted using Matlab R2019b and related toolboxes (MathWork^®^, Natick, Massachusetts): Image Processing™ and Parallel Computing™ toolboxes and Deep Learning™ toolboxes. We also implemented our models using GPU to speed up the training.

### 3.4 Evaluation Metrics

The most widely used evaluation of a classifier performance in the classification task is overall accuracy. However, it has some significant limitations, particularly in the imbalanced datasets.^36^ In particular, when the test set is imbalanced, the trained model tends to favor the majority classes, and the overall accuracy may facilitate overexpressing the classes that lead to highly misleading evaluations in some cases. Therefore, in this paper, we selected the precision, recall, and F1-score to evaluate imbalanced dataset metric.^37^ These metrics were calculated by the follow criteria.

True positive (TP) is the correct classification of the positive class. True negative (TN) is the correct classification of the negative class. False positive (FP) is the incorrect prediction of the positives. False negative (FN) is the incorrect prediction of the negatives. Precision measures the percentage of the positively label samples that are actually positive. Precision is sensitive to class imbalance because it considers the number of negative samples incorrectly labeled as positive:

$$\text{Precision}=\frac{\mathrm{TP}}{\mathrm{TP}+\mathrm{FP}}.$$ (6)

Recall is not affected by imbalance because it is only dependent on the positive group. Recall does not consider the number of negative samples that are misclassified as positive, which can be problematic in problems containing class imbalanced data with many negative samples:

$$\text{Recall}=\frac{\mathrm{TP}}{\mathrm{TP}+\mathrm{FN}}.$$ (7)

The F-measure or F1-score combines precision and recall using the harmonic mean to adjust the relative importance of precision versus recall:

$$F1\text{-score}=2\frac{\text{Precision}\times \text{Recall}}{\text{Precision}+\text{Recall}}.$$ (8)

## 4 Results

The ResNet-2D classification results of fine-tuning parameters at different levels are shown in Table 1. The pr=1 represents fine-tuning all parameters before this node (as can be seen in Fig. 3). To keep consistent for all experiment conditions, no data augmentation is shown in Table 1. The result shows that the higher the F1-score, the more the fine-tuning parameter layers. This result is consistent with a previous study.^28^

**Table 1:** Fine tuning of different weight layers. pr denotes the point of weight freezing in the network (see Fig. 3).

	Recall (mean ± std %)	Precision (mean ± std %)	F1-score (mean ± std %)
pr=1	96.6±6.2	82.9±9.1	81.2±5.1
pr=2	95.4±8.3	82.8±9.1	80.7±5.0
pr=3	97.0±8.3	81.6±9.0	79.6±4.8
pr=4	96.2±9.0	81.9±8.4	78.5±4.9
All	97.3±6.1	82.5±8.4	82.3±5.5

To investigate the effect of imbalance dataset, we not only compared the one-phase and two-phase training but also set two other contrastive experiments, with and without data augmentation. The comparison results are shown in Table 2, which was based on the fine-tuning parameter pr=4. In both methods, the data augmentation and two-phase training improved the classification results. Comparatively, it was found that the two-phase training method was slightly better than the data augmentation method for the performance improvement.

**Table 2:** Results for plaque classification using the ResNet-2D model. One-phase represents using the original data distribution as model inputs.

	ResNet-2D	Recall (mean ± std %)	Precision (mean ± std %)	F1-score (mean ± std %)
No data aug.	One-phase	97.3±6.1	82.5±8.4	82.3±5.5
	Two-phase	98.8±5.2	82.4±8.4	84.9±4.8
Data aug.	One-phase	95.3±6.0	85.4±7.9	85.1±4.9
	Two-phase^a^	97.4±3.8	84.0±7.0	90.2±4.8

a The classification result was the best.

**Fig. 4:**
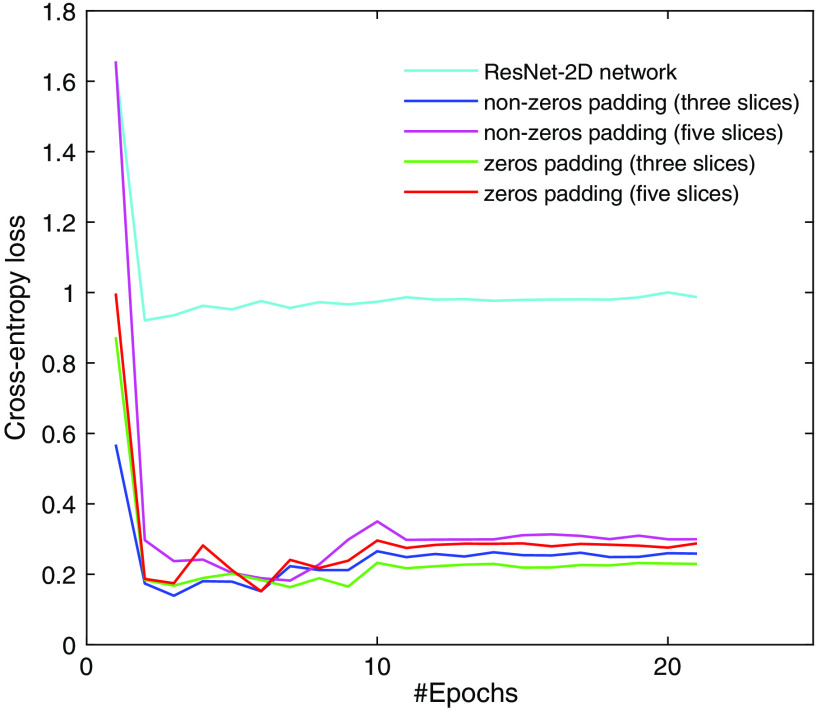
Comparisons of trainings with transfer learning between ResNet-2D and ResNet-3D models in terms of cross-entropy loss (means) on the OCT pullback dataset. Note that the metrics were better when the loss was smaller.

As described in the previous section, we initialized ResNet-3D model parameters by the trained ResNet-2D model (superscript a in Table 2). Here, we compared ResNet-2D and ResNet-3D models convergence using cross-entropy loss on the validation set (Fig. 4). As can be seen, both models converged quickly, which indicated that the model parameters were suitable. In addition, a considerable gap was observed between the ResNet-2D model and the ResNet-3D model, which implied that the ResNet-3D model performed better than the trained ResNet-2D model. Although the loss curves fluctuated greatly when the epoch was less than 10, it became stable when the epoch was more than 15.

The impact of the number of input slices on the ResNet-3D model performance was investigated. The classification results of different methods are shown in Table 3. For both non-zeros padding and zeros padding, a similar phenomenon was observed in terms of the F1-score performance. The five slices achieved the best result followed by the three slices and seven slices. Moreover, majority voting obtained the best results not only for the zeros padding but also for the non-zeros padding, respectively. The performance for zeros padding was better than non-zeros padding with the same number of input slices.

**Table 3:** Results for the ResNet-3D model, which was initialized by trained ResNet-2D model with non-zeros padding and zeros padding.

	ResNet-3D	Precision (mean ± std %)	Recall (mean ± std %)	F1-score (mean ± std %)
Non-zeros padding	ResNet-3D (three slices)	89.1±3.8	87.4±6.9	88.4±5.7
	ResNet-3D (five slices)	97.2±3.6	87.0±6.8	92.6±5.9
	ResNet-3D (seven slices)	95.3±2.4	86.1±6.5	90.9±5.2
	Majority voting^a^	89.0±2.2	99.0±5.4	94.3±4.5
Zeros padding	ResNet-3D (three slices)	98.2±3.8	86.3±6.1	92.6±5.3
	ResNet-3D (five slices)	90.1±3.3	88.8±6.1	94.3±5.0
	ResNet-3D (seven slices)	96.8±2.2	87.3±6.0	91.5±4.2
	Majority voting^b^	96.9±1.3	97.7±3.4	96.1±3.4

a The classification result was the best of the non-zeros padding.

b The classification result was the best of the zeros padding.

**Fig. 5:**
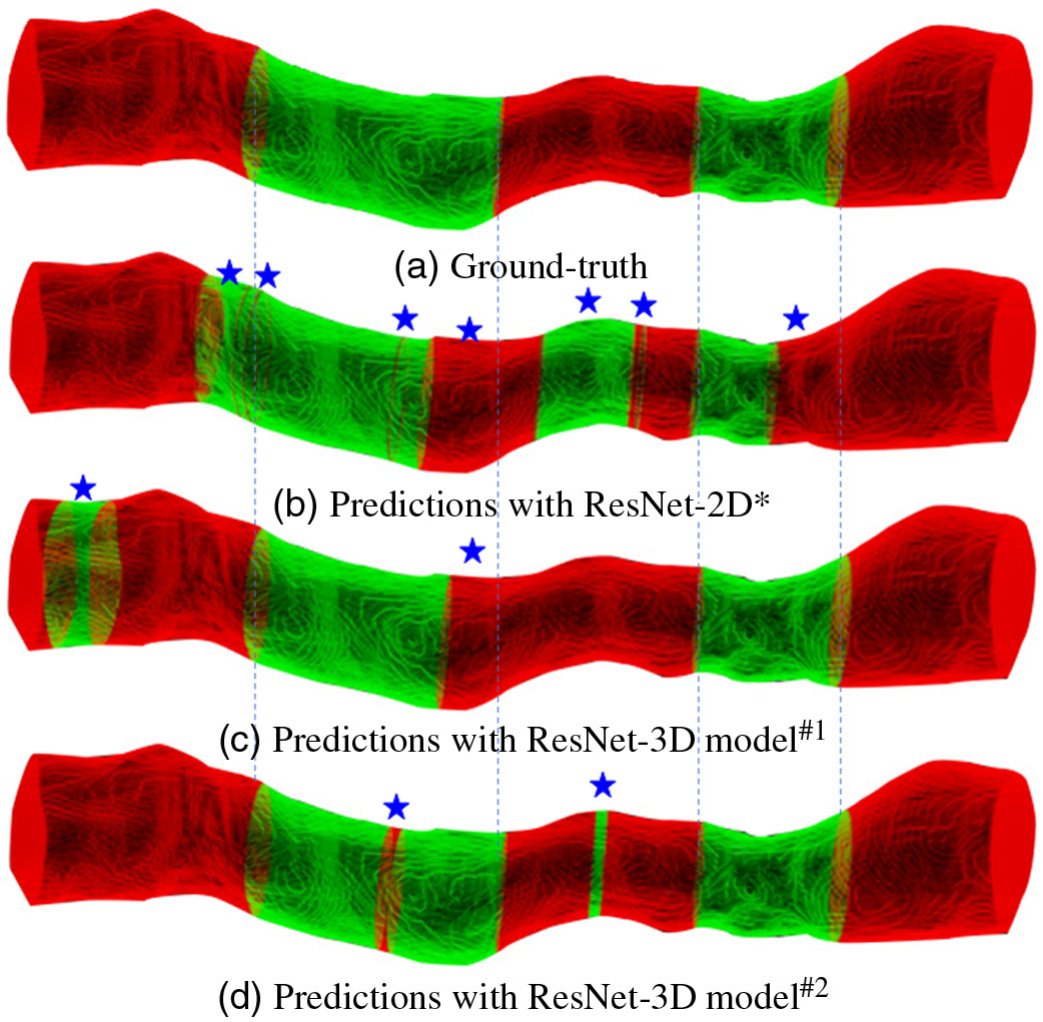
Qualitative results of a pullback using different models. (a) The original pullback with ground-truth labels; (b) predicted results of ResNet-2D model along the pullback; (c) predicted results of ResNet-3D model of non-zeros padding along the pullback; (d) predicted results of ResNet-3D model of zeros padding along the pullback. The red color indicates regions without calcified plaque and the green color indicates regions of calcified plaque.

Figure 5 shows a 3D visualization of the classification results of ResNet-2D model (superscript a in Table 2) and ResNet-3D models (superscript a and b in Table 3) on the pullback level. Compared to the ResNet-2D, the misclassification rate of the ResNet-3D dramatically decreased. In addition, the misclassification results of ResNte-3D models mainly concentrated in the consecutive slices.

The recently published state-of-the-art method by Gessert et al.^21^ analyzed the different transfer learning strategies and network architecture and demonstrated that two-path architecture could provide a more effective feature differentiation. In addition, their study also demonstrated that pr=3 was the optimal option compared to the other stages. Table 4 shows that the comparison of the ResNet-3D model with the two-path model on the OCT data. The advantages of our method are clearly demonstrated.

**Table 4:** Comparison of our implemented method with the method by Gessert et al.^21^ on our dataset.

ResNet-3D	Precision (mean ± std %)	Recall (mean ± std %)	F1-score (mean ± std %)
Zeros padding	96.9±1.3	97.7±3.4	96.1±3.4
Gessert et al.^21^	92.3±4.2	83.7±7.0	90.1±6.1

## 5 Discussion

This study presented ResNet-3D CNN models for an automatic classification of plaque calcification from OCT pullback dataset. The benefits of the transfer learning strategies included a decrease in the computational cost as well as the number of trainable parameters. We proposed an efficient solution for processing large image dataset by adopting the trained parameters based on the ResNet-2D model as the initialization parameters of the ResNet-3D models, alleviating one of the main computational limitations of a 3D CNNs. Furthermore, we also considered the majority voting as a postprocessing step to refine the network’s output, a method that has also shown its advantage for processing 2D images.^38^ It is important to note that the entire process was fully automatic, and no manual intervention and complicated image preprocessing algorithm were involved. In addition, our work could be expanded into a detailed classification system for the clinical classification of plaque morphology in coronary disease, and it may lead to a number of possibilities for future online clinical support system.

The results in Table 1 show that there is a difference in classification performance between both scenarios that full fine-tune network achieves a better classification performance. These results are consistent with the previous research^28^ that suggests that if the target domains are significantly different from source domains, full fine-tune should be performed. In addition, the classification performance of the two-phase method is better than the data augmentation, whereas the time required for the two-phase training is more than data augmentation. In the future, more optimization experiments should be executed to compare them. The results in Table 3 show that the classification performance of zero padding was generally better than the non-zeros padding. The possible reason may be that the adjacent slices provide the complementary information in the ResNet-3D models. In addition, we considered 3, 5, and 7 consequent slices as the ResNet-3D models input. The results suggest that it is better to refer to the five consecutive slices when we label the OCT images. Moreover, it would also be helpful to combine complementary methods through ensemble learning for a best performance.^39^^,^^40^ A majority voting improved the classification result up to 10% for ResNet-2D model, 3% and 5% for non-zeros padding and zeros padding for ResNet-3D. In future research, more research could be extended to investigate the impact of the number of slices on network performance.

The generic nature of our methods allows its straightforward application to different lesion classification tasks without major adaptations. To our knowledge, ResNet-3D network combined with majority voting achieved the highest classification accuracy on an OCT pullback. Gessert et al.^21^ reported that the best classification accuracy, sensitivity, and specificity were 92%, 91%, and 93%, respectively. Table 4 shows that the ResNet-3D model outperforms their two-path model. It is important to note that their work focused only on images excluding stents, whereas our study has shown capable of classifying an entire OCT pullback. Moreover, the work described in this article may be extended to other applications for future research. For example, we can start with three 2D networks trained for lateral and coronal sections, and then initialize the 3D network. Also, we could consider 3D voxels segmentation and use the spatial information between adjacent slices to segment OCT voxels.

Although neural networks seem promising for OCT image classification, further work is required to make the inference process more interpretable. This would improve our understanding when the network fails, an important aspect in biomedical applications. Although the ResNet-3D network needed one-hour training time, an optimal and simple network may further speed up the convergence.

## 6 Conclusion

A Resnet-2D model based on each OCT slice was implemented to meet the challenges of automated classification of plaque calcification in coronary artery. Then, we established an automatic classification model on the OCT pullback level by expanding the ResNst-2D model to the ResNet-3D model. We compared non-zeros padding and zeros padding methods to convert the 2D convolutional filter to 3D. The result demonstrated that zeros padding was more suitable for the ResNet-3D model. We further implemented a majority voting algorithm by incorporating a contextual slice integration scheme for accurate lesion classification. Our study demonstrated that ResNet-3D models with effective training mechanisms could be employed to solve complicated medical image classification problems, even with a limited training dataset. Further investigations can be focused on integrating supervised information into our networks to further enhance the discrimination capability and explore more applications.
